# Atypical haemolytic uremic syndrome with posterior reversible encephalopathy syndrome in an adolescent: a rare case report

**DOI:** 10.11604/pamj.2022.43.71.36406

**Published:** 2022-10-11

**Authors:** Sneha Kenjale, Keta Vagha, Jayant Vagha, Ashish Varma, Lavanya Ram Krishnan, Dewang Ghode

**Affiliations:** 1 Pediatrics Department, Jawaharlal Nehru Medical College, Sawangi (Meghe), Wardha, Maharashtra, India

**Keywords:** Posterior encephalopathy, renal failure, renal hypertension, schistocytes, case report

## Abstract

Atypical hemolytic uremic syndrome (aHUS) is a group of disorders that affect kidneys which is rare type of HUS that differs from classical hemolytic uremic syndrome (HUS) by absence of prodromal phase consisting of episodes of diarrhoea due to preceding shiga toxin E. coli (STEC-HUS) infection and is 5% of all HUS cases. Approximately 50% cases present with clinical triad of hemolytic anemia, thrombocytopenia and renal insufficiency. However, it can have unusual clinical features in form of central nervous system involvement. This case, of a 15-year-old Indian boy, is one such rare presentation of atypical haemolytic uremic syndrome associated with posterior reversible encephalopathy syndrome (PRES), or reversible posterior leukoencephalopathy syndrome (RPLS) who presented with anaemia, anasarca, papilledema, hypertension, episodic seizures and significant magnetic resonance imaging (MRI) brain findings. We report this uncommon combination of two syndromes to provide useful insight for clinicians to approach and diagnose such presentation in paediatric patients.

## Introduction

Atypical haemolytic uremic syndrome (aHUS) is a group of disorders that is one of the causes of acute kidney injury (AKI) in children. It differs from classical HUS by absence of any prodromal period and preceding shiga toxin *E. coli* (STEC-HUS) or shigella infection [1]. It can be familial or sporadic and majority have bad prognosis. Prevalence of aHUS in people of age 20 years or less is found to be in range of 2.2 to 9.4 million and is equal in males and females [1]. It presents with triad of thrombocytopenia, microangiopathic haemolytic anaemia, renal insufficiency and consequently, hypertension due to renal failure.

Posterior reversible encephalopathy syndrome or RPLS is mainly a radiological diagnosis which occurs due to hypertension, malignancy, collagen vascular diseases, sepsis and HUS [2]. It usually presents with headache, blurring of vision, cortical blindness, confusion or seizures [3]. Neuroimaging of PRES shows characteristic parieto-occipital white matter edema and hyperintensities [4]. Reversible posterior leukoencephalopathy syndrome is reversible but lesions become irreversible in long run, causing chronic epilepsy or death [2,5]. This case holds rarity because of its unusual combination of two syndromes together, that is, aHUS and PRES in a paediatric patient, which are fatal enough but are treatable when diagnosed and managed early. With this background, we report a case of a 15-year-old boy who presented with complaints of swollen limbs, hematemesis, hypertension and convulsions who was diagnosed as a case of PRES secondary to aHUS. However, patient´s age and financial status poses a great challenge in management of these disorders.

## Patient and observation

**Patient information:** a 15-year-old Indian male, accompanied by mother. He had no significant family history.

**Clinical findings:** patient presented to us with swollen limbs and face since seven days, two episodes of hematemesis since four days and one episode of generalised tonic clonic convulsion in the morning on the day of admission. The patient had developed swelling over his face initially and then gradually involved whole of the body in the duration of these seven days. There was no history of loose stools, cough and cold or decreased urinary output.

**Past interventions and outcomes:** he was initially hospitalised in another hospital where he had undergone the initial treatment and the following course. On examination, pulse was 98 beats per minute, blood pressure was 122/80 mmHg, respiratory rate was 23 cyles per min and was afebrile with no systemic abnormalities. His baseline blood panel showed hemoglobin- 5.9 gm/dL, total leucocyte count- 7200/cumm, Platelets- 2.4 lacs/cumm, urea- 148 and creatinine- 2.4 mg/dL. He was given single unit of packed red cell. His blood pressure showed gradual increase and was at the 90^th^ centile. On regular monitoring the blood pressure readings were around 160/110 mmHg, therefore injection labetalol was given for stage II hypertension and was sent to our centre for further management.

**Timeline of current episode:** when he was brought to our institute, he was in active generalised tonic-clonic type of convulsion which was stopped by midazolam injection and then patient was shifted to paediatric intensive care unit.

On examination of the patient, his pulse was 58 beats per minute with respiratory rate of 22 breaths per minute, blood pressure of 180/120 mmHg and Spo_2_of 97% on room air. He was drowsy, brisk deep tendon reflexes and no other systemic abnormality. He was given ceftriaxone injection along with intravenous fluids, levetiracetam and injection sodium nitroprusside.

**Figure 1:**
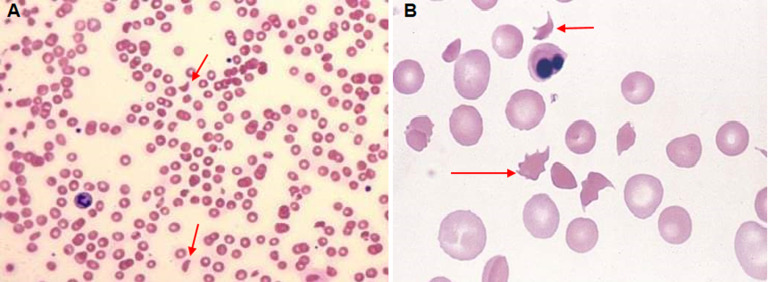
A) peripheral blood smear of the patient, arrow mark is showing schistocytes and hypochromic red blood cells suggestive of hemolytic anemia (10X, hematoxylin-eosin stain); B) peripheral blood smear of the patient, arrow mark is showing schistocytes (oil immersion 100X, hematoxylin-eosin stain) suggestive of hemolysis

**Figure 2:**
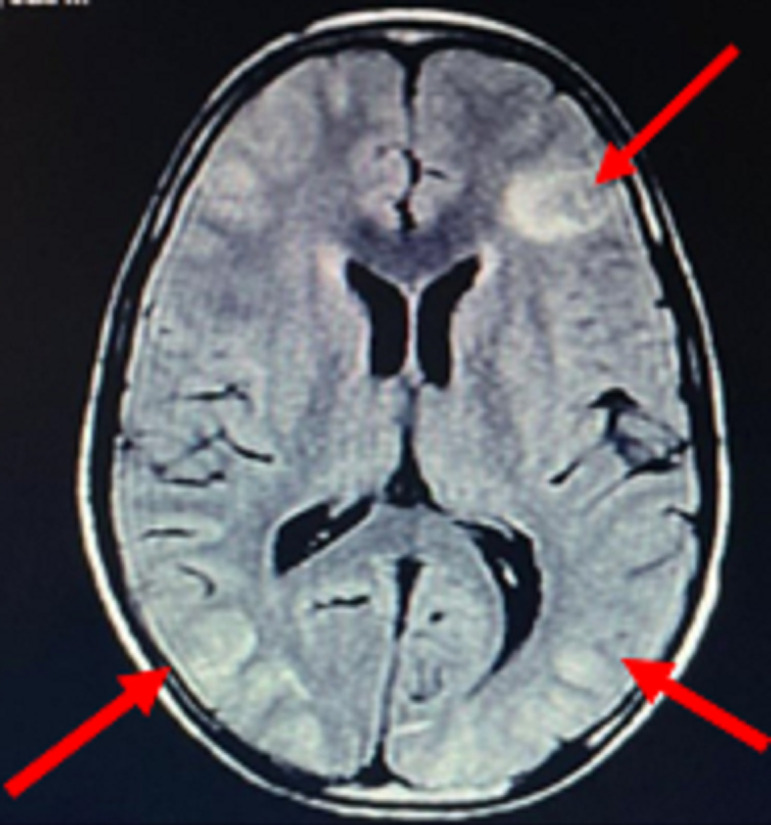
magnetic resonance imaging of brain showing posterior reversible encephalopathy, arrows showing oedematous and hyperintense gyri in bilateral parieto-fronto-occipital region on T2/flair suggestive of posterior reversible encephalopathy syndrome

**Diagnostic assessment:** his complete blood, urine and serological investigations were done sequentially to monitor response to treatment (Table 1). His peripheral smear on day 1 and day 3 of admission showed no abnormality but on day 4, schistocytes (Figure 1) were detected suggesting us diagnosis of HUS as there was gradual fall in the platelets as well as deterioration is renal function. His C3 levels were also low suggestive of hypocomplementemia which also pointed us towards the diagnosis of HUS. As there was no prodromal phase and there was no thrombocytopenia at the onset of the disease process, he was diagnosed as a case of aHUS. Magnetic resonance imaging brain showed hyperintense and oedematous gyri in bilateral parieto-occipital region in T2 flair, suggestive of PRES or reversible posterior leukoencephalopathy syndrome (RPLS) as seen in Figure 2. His electroencephalogram was also done which showed generalised epileptogenic activity. Fundoscopy showed papilledema in left eye and later both eyes.

**Table 1:** sequential lab investigations done in the patient

Laboratory findings of patient	Day 1	Day 3	Day 4	Day 5	Day 6	Day 7	Day 8	Day 14	Day 28 (follow-up after 2 weeks)
Hb (g/dl)	10.9	12.4	10	9.4	10.7	10	9.3	7.8	10.3
Platelets (lakhs/cumm)	2.25	0.83	0.47	0.57	0.64	0.69	1.06	3.47	3.5
Urea (mg/dl)	126	107	114	116	94	110	105	105	64
Creatinine (mg/dl)	2.7	1.9	2.0	2.2	1.6	1.4	1.4	1.4	0.8

**Diagnosis:** laboratory and imaging investigations altogether helped to establish diagnosis of atypical hemolytic uremic syndrome along with posterior reversible encephalopathy syndrome.

**Figure 3:**
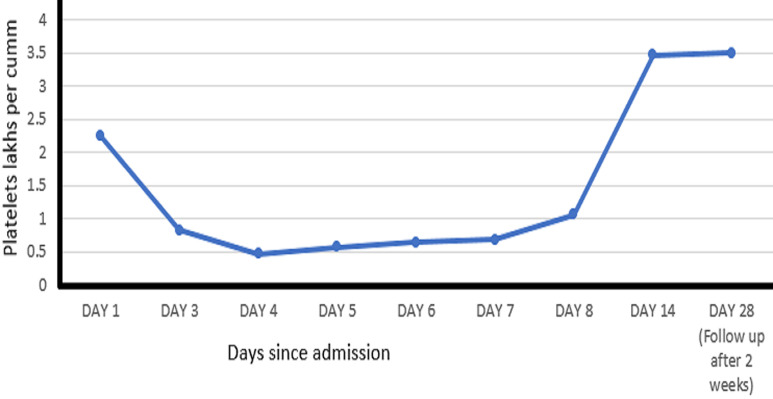
a scatter diagram suggesting patient recovery and follow-up, it shows gradual increase in platelets as per day of admission suggesting improvement in patient condition

**Therapeutic interventions:** plasmapheresis and renal biopsy was the next step and patient was advised the same but could not be done due to limited financial aids. Patient was stabilised with the help of medicines and a single unit of fresh frozen plasma. He was started on oral steroids at 2mg/kg/day. The patient showed gradual improvement as the blood pressure started to normalise and also the renal function and the platelets in the duration of 14 days and was advised gradual tapering of the oral steroid. He was then followed up after one month which showed the blood pressure to be 120/68 mm Hg, the platelets were improved to 3.5 lacs/cumm (Table 1, Figure 3) and renal function test showed urea- 54 mg/dL and the creatinine- 1.0 mg/dL.

**Follow-up and outcome of interventions:** he was advised for routine follow-up to completely taper off the oral steroids. He was followed up after two weeks of his discharge and his blood pressure was 108/60 mmhg which was normal for his age and height. Also, there was improvement in blood parameters, hemoglobin- 10.3 gm/dl, platelets- 3.5 lacs/cumm (Figure 3) and renal function as the renal function tests showed- urea- 64 mg/dL, creatinine- 0.8, sodium- 140 mg/dL and potassium 3.8 mg/dL. The oral steroids were weaned off gradually over the next four weeks.

**Informed consent:** the parents of the patient were well informed and gave us written consent to publish this case report along with any relate images.

## Discussion

Amongst the uncommon types of thrombotic microangiopathy, atypical hemolytic uremic syndrome consists of 5-10% cases of total HUS cases and is a well-known cause of acute renal failure in children. However, it is rarely found in association with posterior reversible encephalopathy syndrome (PRES) and our case is one of them.

The presentation is acute in about 20% of cases and severity of clinical features correlate with the extent of microvascular injury. Pallor and low urine output is present commonly, which is suggestive of anemia and renal insufficiency, respectively [6]. As microvascular lesion affects interlobular arteries in kidneys, hypertension of moderate to severe range can be seen. Apart from this, other signs and symptoms suggestive of renal insufficiency like proteinuria (history of frothy urine), hematuria and azotaemia are sometimes found [7]. Consequently, patient of renal failure lands into renal hypertension leading to posterior reversible leukoencephalopathy. Broad spectrum of presentations and varied prognoses of atypical HUS are possible. A case report by Kedsatha *et al*. had typical presentation of sudden weight gain of 3 kilograms with anemia due to severely rising oedema in a 13-year-old girl of Laos. She was presumptively treated for rapidly progressive glomerulonephritis, secondary to post streptococcal glomerulonephritis as anti-streptolysin O (ASO) titres were positive but patient couldn´t survive due to rapid deterioration of renal function [1]. Whereas another report by Fallahzadeh *et al*. had relatively better prognosis. Absence of common features like hypertension, edema and oliguria was noted in 9-year-old boy with aHUS who was infected with Ebstein Barr virus. Remission was achieved within 12 months with help of dialysis. Here, disease had good prognosis even with absence of common signs of atypical HUS [8].

Apart from the typical triad of atypical HUS, nervous system involvement in form of PRES is rare to see but not impossible. It can clinically present as headache, confusion, blurring of vision, cortical blindness or seizures and radiologically, as hyperintensities in parieto-occipital region [3]. Neurologic manifestations, like PRES in our case, is usually is the result of renal hypertension caused due to renal failure. RPLS can have renal aetiologies other than atypical HUS, like in a case series by Ganesh *et al*. two 12-year-old patients developed PRES due to focal segmental glomerulosclerosis and steroid depended nephrotic syndrome, respectively [4]. Another three paediatric cases reported by Emeksiz *et al*. had been diagnosed with lupus nephritis, acute glomerulonephritis and Henoch Schoenlein purpura nephritis, respectively, and all of them presented with seizures due to PRES [2].

Recurrent PRES was also reported in a 2-year-old child with non-communicating hydrocephalus due to renal failure and etoposide, an anti-cancer medication given for macrophage activation syndrome [3]. In management of atypical HUS and its neurological manifestations, plasmapheresis or plasma exchange and infusion remains mainstay treatment [6]. It helps to achieve remission as seen in case of 15-year-old Srilankan girl reported by Basnayake *et al*. with high blood pressure of 180/100, pulmonary haemorrhage and severe sepsis and was diagnosed with atypical HUS. Her lab investigations showed thrombocytopenia and schistocytes in peripheral blood smear, hypocomplementemia and raised lactate dehydrogenase (LDH) levels, which was also seen in our case that gave definitive diagnosis. Complete remission was achieved after treatment of 30 months with plasmapheresis [7]. A newer drug, eculizumab is becoming gold standard treatment of atypical HUS now and is replacing plasmapheresis. This drug is a high affinity anti-C5 monoclonal antibody that stops disease progression by aborting abnormal alternate complement pathway. However, this drug is not much affordable and available in Indian setting. Cases of two paediatric patients reported by Gulleroglu *et al*. had severe symptoms like, seizures, vision loss, loss of balance and nystagmus, which drastically improved just within 24 hours of eculizumab administration [6]. Paediatric patients, out of all, need immediate and effective treatment but in our case limited financial aids posed great difficulty to provide definitive management in form of plasmapheresis.

Our case of a 15-year-old male, who presented with 5.9 gm/dl of haemoglobin with anasarca and deranged renal function tests, was diagnosed with aHUS associated with RPLS or PRES. Creatinine and urea was significantly raised along with schistocytes in peripheral blood smear. Significant antibodies were absent with negative blood and urine tests. Presence of hypocomplementemia suggested atypical haemolytic uremic syndrome. Typical radiological evidence of hyperintense and oedematous gyri in parieto-occipital region suggested posterior reversible encephalopathy syndrome which was the result of hypertension due to renal failure. Diagnosis of Atypical hemolytic uremic syndrome with posterior reversible encephalopathy syndrome was thus established.

## Conclusion

Atypical haemolytic uremic syndrome along with its neurological manifestation in the form of posterior reversible encephalopathy syndrome is a rare and fatal combination to find in a pediatric patient. However, early diagnosis with the help of serological and radiological modalities along with oral steroids and symptomatic treatment gave better prognosis in this child, despite of limited financial aid. This case sheds light on the need of early diagnosis and prompt management that is essential in such life-threatening cases, considering the challenges posed, to provide better quality of life to patient.
